# STAMPS: development and verification of swallowing kinematic analysis software

**DOI:** 10.1186/s12938-017-0412-1

**Published:** 2017-10-17

**Authors:** Woo Hyung Lee, Changmook Chun, Han Gil Seo, Seung Hak Lee, Byung-Mo Oh

**Affiliations:** 1 Department of Rehabilitation Medicine, Seoul National University Hospital, Seoul National University College of Medicine, 101 Daehak-ro, Jongno-gu, Seoul, 03080 Republic of Korea; 2 Center for Bionics, Biomedical Research Institute, Korea Institute of Science and Technology, Hwarang-ro 14-gil, Seoungbuk-gu, Seoul, 02792 Republic of Korea; 3 Department of Biomedical Engineering, Seoul National University College of Medicine, 101 Daehak-ro, Jongno-gu, Seoul, 30380 Republic of Korea

**Keywords:** Deglutition, Biomedical phenomena, Kinematic analysis, Software, Reliability and validity analysis

## Abstract

**Background:**

Swallowing impairment is a common complication in various geriatric and neurodegenerative diseases. Swallowing kinematic analysis is essential to quantitatively evaluate the swallowing motion of the oropharyngeal structures. This study aims to develop a novel swallowing kinematic analysis software, called spatio-temporal analyzer for motion and physiologic study (STAMPS), and verify its validity and reliability.

**Methods:**

STAMPS was developed in MATLAB, which is one of the most popular platforms for biomedical analysis. This software was constructed to acquire, process, and analyze the data of swallowing motion. The target of swallowing structures includes bony structures (hyoid bone, mandible, maxilla, and cervical vertebral bodies), cartilages (epiglottis and arytenoid), soft tissues (larynx and upper esophageal sphincter), and food bolus. Numerous functions are available for the spatiotemporal parameters of the swallowing structures. Testing for validity and reliability was performed in 10 dysphagia patients with diverse etiologies and using the instrumental swallowing model which was designed to mimic the motion of the hyoid bone and the epiglottis.

**Results:**

The intra- and inter-rater reliability tests showed excellent agreement for displacement and moderate to excellent agreement for velocity. The Pearson correlation coefficients between the measured and instrumental reference values were nearly 1.00 (P < 0.001) for displacement and velocity. The Bland–Altman plots showed good agreement between the measurements and the reference values.

**Conclusions:**

STAMPS provides precise and reliable kinematic measurements and multiple practical functionalities for spatiotemporal analysis. The software is expected to be useful for researchers who are interested in the swallowing motion analysis.

**Electronic supplementary material:**

The online version of this article (doi:10.1186/s12938-017-0412-1) contains supplementary material, which is available to authorized users.

## Background

Swallowing impairment is a common complication in various geriatric and neurodegenerative diseases and accounts for over 15% of cases in the elderly population [1, 2]. It has been gradually recognized as a serious problem in the society in accordance with the aging trend in recent years. Identifying the dynamic features of swallowing and the main mechanism of aspiration is essential in establishing strategies to enhance recovery from swallowing impairment. Swallowing kinematic analysis (SKA) is considered one of the most important methods for the quantitative evaluation of swallowing. SKA has been conducted to investigate the motions of oropharyngeal structures in spatiotemporal aspects [3]. The videofluoroscopic swallowing study (VFSS) visualizes the dynamic motions of the superficial and deep structures of the oropharyngeal region. Hence, most of the analyses have been conducted using real-time fluoroscopic images obtained from the VFSS [4].

Although swallowing motion has been one of the minor fields compared to other biomechanical analysis of human movements such as gait, grasping or spinal kinematics, it has attracted increasing research interest in recent years along with higher demands in clinical practice. However, analysis software that can be used by swallowing researchers is still rare, and in most cases, generic motion analysis software has been used. Commercial software may restrict wide application with expensive license fees despite its high performance and free software that is not optimized for motion analysis of swallowing can be insufficient to enable to conduct researches efficiently and effectively [5–8]. For research purposes, only two studies were reported to develop swallowing motion software that can deal with only simple temporal parameters (e.g., oral and pharyngeal transit time) measured by counting frames [9, 10]. In these environments, it seems to be also unfavorable for clinicians in clinical practice to assess swallowing function in patients with swallowing impairment using the computer-based approach. It is necessary to develop open-source SKA software that can be used efficiently and practically for research or clinical application.

The present study introduces a novel MATLAB-based, open-source SKA software, called spatio-temporal analyzer for motion and physiologic study (STAMPS) and proves its validity and reliability. This software was developed to acquire and process the spatio-temporal data from VFSS video and visualize swallowing motions using diverse graphs for intuitive interpretation. The software is available at https://github.com/cmookj/stamps, and runs on Mac, Linux, and Windows only if MATLAB with image processing toolbox is installed.

### Application of the STAMPS software in swallowing motion analysis

The current study introduced the novel software, STAMPS, to support the analysis of swallowing motions. This software was designed to analyze simultaneous movements of various structures during swallowing. Using the software, multidimensional spatiotemporal data of each structure generated in the swallowing motion can be efficiently obtained, calculated, and displayed. The swallowing motion is analyzed in a two-dimensional space because the two-dimensional images are obtained using the videofluoroscopy which is one of the standard evaluation tool for swallowing function in a clinical setting. In most of the swallowing structures and food bolus, displacement and velocity in the vertical/horizontal directions are regarded as important: the motion in anterior/superior direction followed by posterior/inferior direction of the hyoid bone, larynx, and arytenoid, dilatation of the upper esophageal sphincter, and passage in posterior/inferior direction of food bolus. Angular displacement and velocity is critical only for the epiglottis, which has a role of covering the larynx to prevent the leakage of food bolus. For each structure, it may be appropriate to analyze the linear motion in the coordinate axis or the angular motion for a specific axis and this software allows the motion data to be calculated considering the characteristic motion of such a structure. This software has a set of functionalities for temporal and spatial analyses based on the previous researches [5, 6, 12]: the setting of the local coordinate system, determination of the spatio-temporal parameters for specific target structures, and plotting processes. The sample tests using clinical and instrumental data showed good reliability and validity for motion analysis. The datasets analyzed during the current study are not publicly available due the privacy of the patients but can be available from the corresponding author on reasonable request. Considering that SKA has been one of the minor fields in motion analysis and software analysis tool has been rarely developed, the software developed in this study is expected to contribute to the convenience and efficiency for the researchers in this field.

This software not only allows researchers to acquire and analyze time-series motion data easily and efficiently, but also can be used by clinicians to analyze the patient’s condition and determine treatment methods in clinical practice. The STAMPS provides information of the patient-specific swallowing mechanics of diverse structures simultaneously. It can be used to analyze the mechanism of treatment effect comparing the motion data before and after the treatment or to identify the gradual aggravation of swallowing function in patients with chronic progressive diseases such as Parkinson’s diseases or amyotrophic lateral sclerosis. The intuitive user interface can be beneficial for clinicians who are not familiar with the programming language to obtain the results of motion analysis with diverse types of graphs. The authors expect STAMPS can be a valuable tool providing precise and reliable kinematic measurements to help researchers and clinicians efficiently and effectively perform SKA and provide an understanding of the swallowing motions.

## Methods

### Coordinate systems

**Fig. 1:**
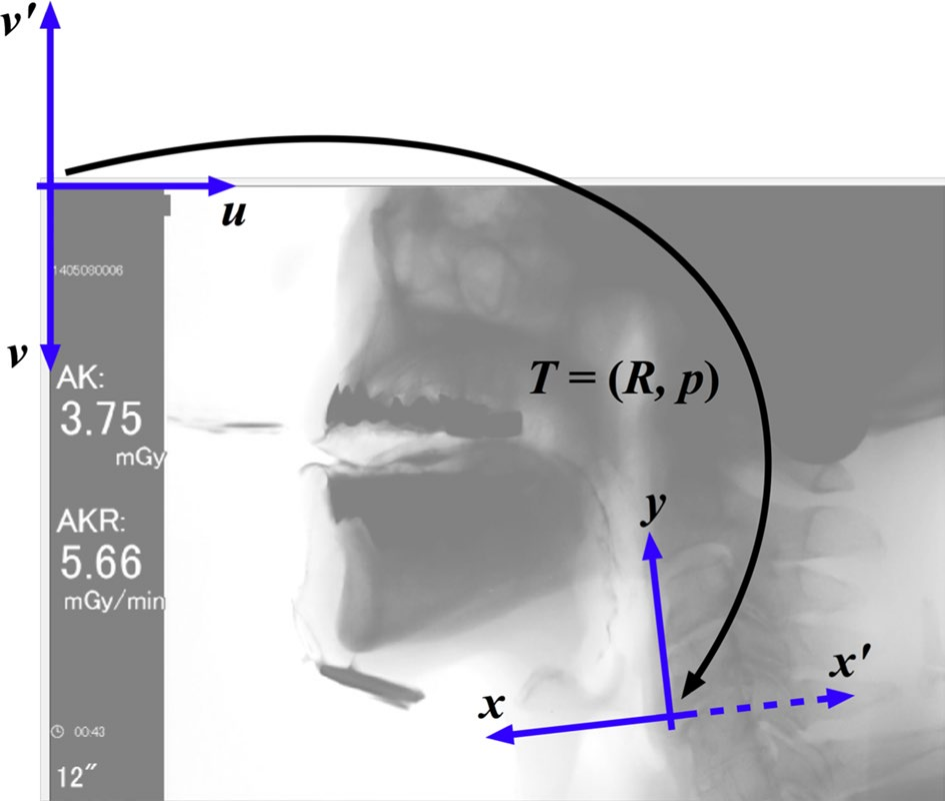
A representative videofluoroscopic image and the coordinate systems

STAMPS defines a local coordinate system for each image. The 2nd and 4th cervical vertebral bodies (i.e., C2 and C4, respectively) specify the coordinate frame. The origin is at the anteroinferior vertex of the C4. The vertical axis connects the origin to the anteroinferior vertex of the C2, while the horizontal axis stretches out from the origin to the left of the vertical axis. This horizontal axis is opposite in direction to that of the conventional coordinate systems. Note that this local coordinate frame is not fixed in space. It moves along the cervical vertebral bodies (Fig. 1). The local coordinate system of STAMPS is derived from previous studies [5, 6].

The MATLAB image processing toolbox treats an image as a grid of discrete elements. Each element is accessible by pixel indices $\left( {u, v} \right)$, where *u* and *v* increase to the right and downward, respectively. The coordinate transform from $\left( {u, v} \right)$ to $\left( {x, y} \right)$ is presented as follows considering the increasing directions of the axes of the image and the local frames:

$$ \left[ {\begin{array}{*{20}c} x \\ y \\ \end{array} } \right] = s\left[ {\begin{array}{*{20}c} { - \,1} & 0 \\ 0 & 1 \\ \end{array} } \right]\left[ {\begin{array}{*{20}c} {R^{\text{T}} } & { - \,R^{\text{T}} p} \\ 0 & 1 \\ \end{array} } \right]\left[ {\begin{array}{*{20}c} 1 & 0 \\ 0 & { - \,1} \\ \end{array} } \right]\left[ {\begin{array}{*{20}c} u \\ v \\ \end{array} } \right] = s\left[ {\begin{array}{*{20}c} { - \,R^{\text{T}} } & { - \,R^{\text{T}} p} \\ 0 & { - \,1} \\ \end{array} } \right]\left[ {\begin{array}{*{20}c} u \\ v \\ \end{array} } \right], $$

where $T = \left( {R, p} \right) \in SE\left( 2 \right)$ is the pose of the $\left( {x^{\prime}, y} \right)$ frame with respect to the $\left( {u, v^{\prime}} \right)$ frame, and *s* is a scale factor. The special Euclidean group *SE*(2) is homeomorphic to ${\mathbb{R}}^{2} \times {\mathbb{S}}^{1}$, whose element represents a rigid body motion in a two-dimensional space (Fig. 1). The scale factor is a ratio between the true and observed-in-image lengths of a reference object. The default settings of STAMPS assume a coin with a 24.0 mm diameter.

### File formats, interface design, and functionalities

STAMPS can import all video formats that MATLAB supports including avi, mpg, mp4, m4v, and mov. STAMPS saves all the data related to a mat file for each analysis and exports data into a plain text file to enable further analysis utilizing other tools.

**Fig. 2:**
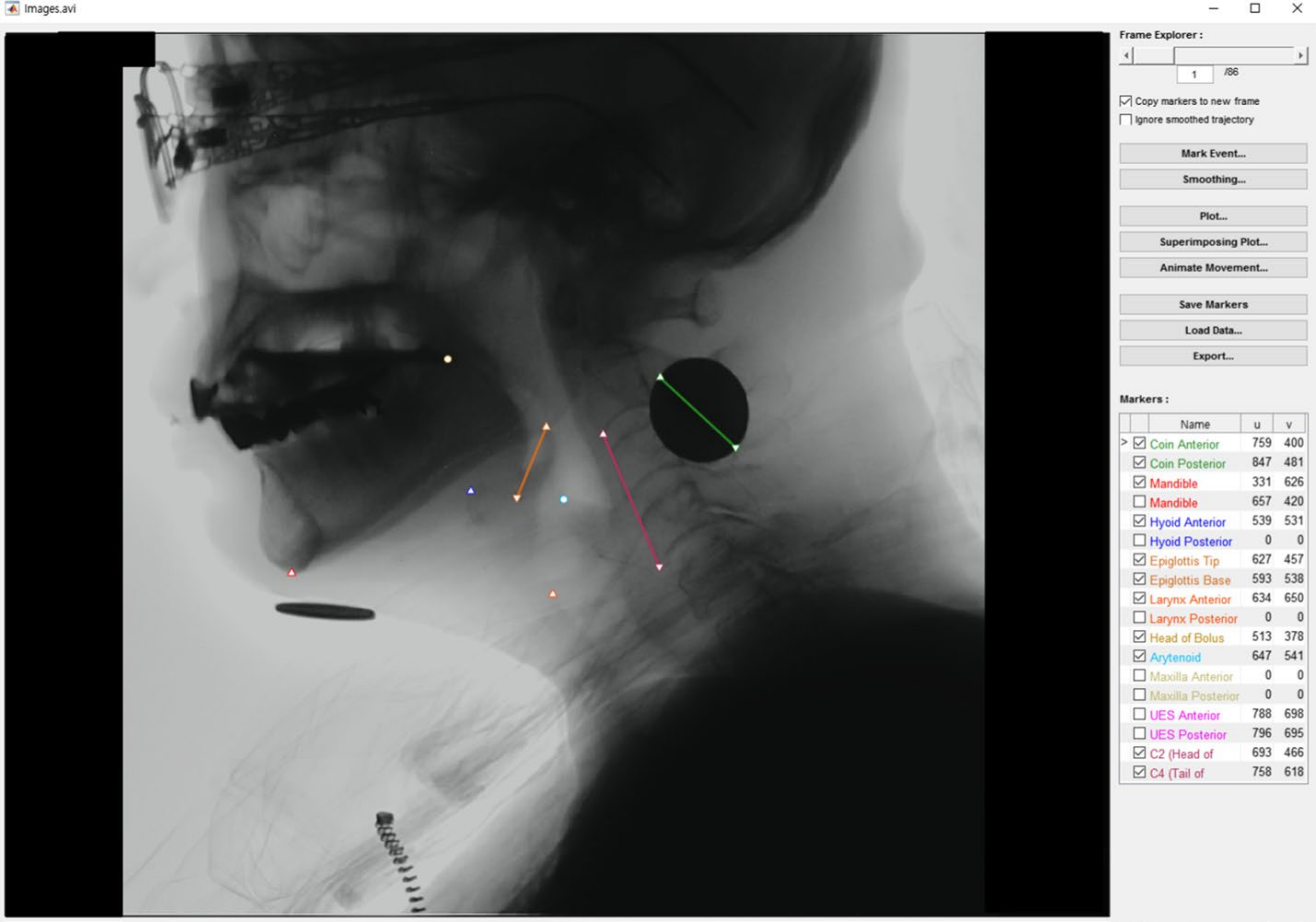
Main interface of the spatio-temporal analyzer for motion and physiologic study (STAMPS)

Intuitiveness and usability are the principles of the interface design of STAMPS (Fig. 2). American architect Louis Sullivan coined a well-known phrase “Form follows function,” which is popular in modernist architecture and industrial design. It insists that the intended function or purpose of an object should determine its shape. The STAMPS interface is the result of a considerate application of the principle. All the controls on the right side of the main window are self-explanatory.

We describe the four main functionalities of STAMPS in this section, namely event recording, raw trajectory marking, smoothing, and visualization.

#### Event recording

The button labeled as “Mark Event…” is used to record specific events, such as the beginning of a motion of an oropharyngeal structure. The current version of STAMPS supports 20 discrete events, which are editable in its preference window. The button opens a dialog window with a table view showing the list of events. A user records an event by selecting the event in the dialog while examining the image frame where the event occurs. The event has an ordinal number of the frame in the video stream. All the supported video formats have their own frame rate (i.e., the number of frames in a second). Calculating the time elapsed between any two events recorded is trivial.

#### Raw trajectory marking

A raw trajectory is the sequence of the positions of a target structure obtained from each frame. The mouse clicks in an image frame define the positional data. A table view can be found on the right side of the image frame. The table lists the target structures relevant to the swallowing analysis. The first column in the table indicates a marker for the next mouse click to specify the position of. The second column shows active markers to track for the analysis. The remaining columns are self-explanatory. Note that the tuples in the table denote the image coordinate system.

STAMPS copies all the current positional data to the new frame when it loads a new image frame. We usually build raw trajectories by marking the targets in the order of time sequence. The common rate of video format frames is 30/s. In other words, the time difference between the consecutive frames is 0.033 s. The oropharyngeal structure motion is often slow, and the differences of the markers between the frames are small or negligible. Thus, copying the current data to a new frame reduces the burden of manual marking.

The default set of target structures for the SKA includes bony structures (hyoid bone, mandible, maxilla, and cervical vertebral bodies), cartilages (epiglottis and arytenoid), soft tissues [larynx and upper esophageal sphincter (UES)], and food bolus.

#### Trajectory smoothing

STAMPS provides most common methods for trajectory smoothing: polynomial curve fitting, moving average, LOWESS (locally weighted scatterplot smoothing), LOESS (generalization of LOWESS), Savitzky–Golay filter, robust LOWESS, and robust LOESS. The choice of the smoothing method depends on the user’s preference and experiences. The simplest methods such as moving average and polynomial curve fitting are suitable in most cases. However, when the classical algorithms do not perform well, the authors recommend more advanced methods such as LOWESS, LOESS, or Savitzky–Golay filter. LOWESS and LOESS build on classical methods such as linear or nonlinear least squares regression in a k-nearest-neighbor-based meta model, and Savitzky–Golay filter, which fits subset of successive adjacent data with a low-degree polynomial, is a generalization of moving average filter. Accordingly, choosing a method for each trajectory is possible in a dialog dedicated for the smoothing. A brief description of the method and the user interfaces to control its parameters is provided.

#### Visualization

**Fig. 3:**
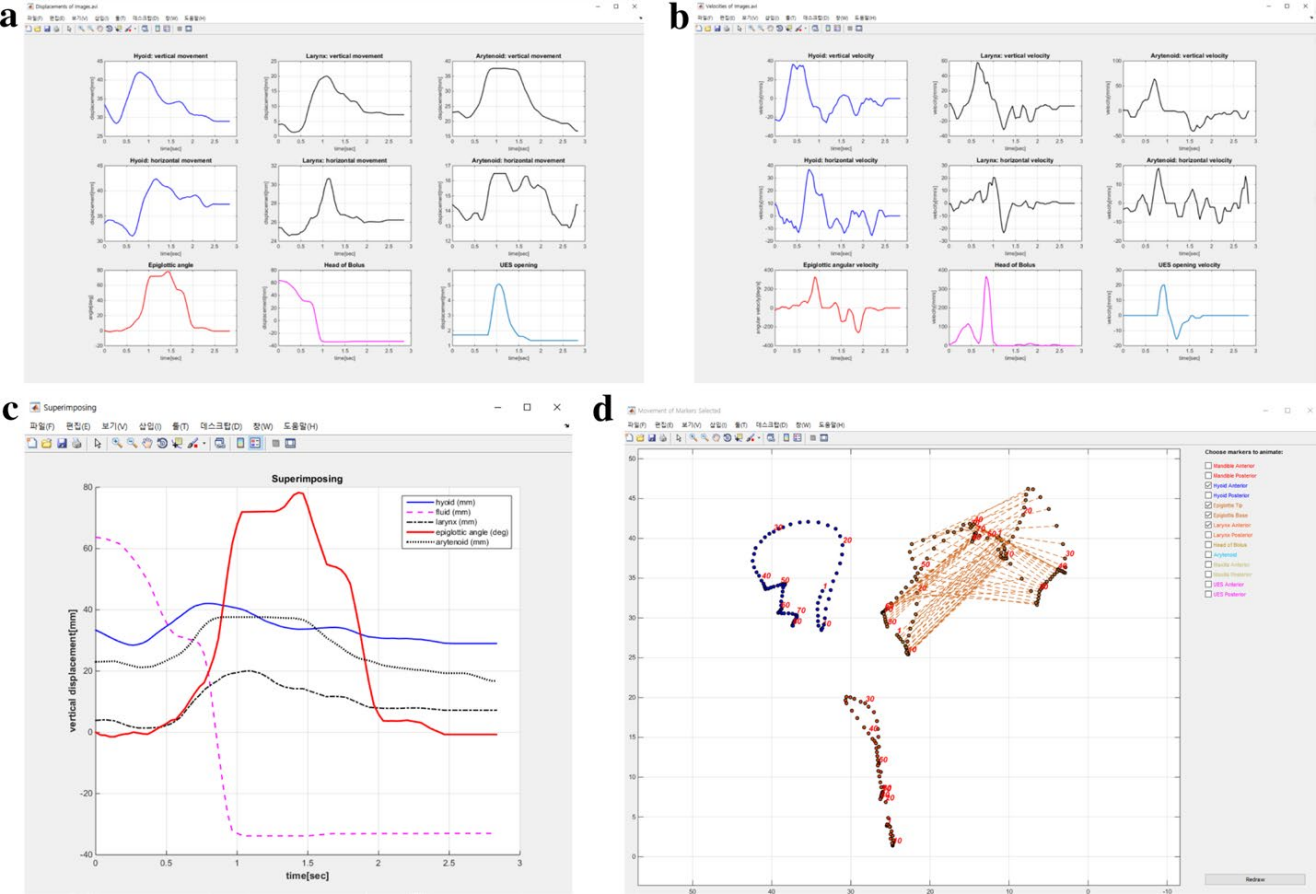
Examples of the visualized graphs for displacement (**a**), velocity (**b**), superimposed vertical displacement (**c**), and animated trajectories (**d**) of the analyzed structures

Three tools are utilized to visualize the marker motion. The first tool aims to plot the displacements and velocities of the key oropharyngeal structures. The default selection includes the hyoid, larynx, arytenoid, epiglottis, bolus, and UES opening. The characteristic motion of each swallowing structure determines the visualization type. For example, STAMPS shows the angular displacement and the velocity for the epiglottis with a folding motion. It also displays the linear displacements and the velocities for other structures, such as the hyoid bone, larynx, and liquid bolus (Fig. 3a, b). STAMPS approximates the velocities by the sequence of the finite differences of the displacement, i.e., given a sequence of positions {*x*(*t*_*i*_)} $\left( {i = 0, \ldots ,n} \right)$, the sequence of velocities {*v*(*t*_*i*_)} is determined by *v*(*t*_0_) = (*x*(*t*_1_) − *x*(*t*_0_))/Δ*t*, $v\left( {t_{i} } \right) = {{\left( {x\left( {t_{i + 1} } \right) - x\left( {t_{i - 1} } \right)} \right)} \mathord{\left/ {\vphantom {{\left( {x\left( {t_{i + 1} } \right) - x\left( {t_{i - 1} } \right)} \right)} {2\Delta t,}}} \right. \kern-0pt} {2\Delta t,}} \;\left( {i = 1, \ldots ,n - 1} \right)$, and *v*(*t*_*n*_) = (*x*(*t*_*n*_) − *x*(*t*_*n*-1_))/Δ*t*, where Δ*t* = *t*_*i*+1_ − *t*_*i*_ is assumed to be constant all over the *i*. The second tool superimposes the vertical displacements of the structures (Fig. 3c) and is useful in recognizing the swallowing patterns because the simultaneous motions of the bolus swallowed are organized [11]. Finally, the third tool animates the motions of the selected markers (Fig. 3d).

### Verification of reliability and validity of the program

For the reliability tests, the swallowing motions were captured from 10 dysphagia patients (mean age, 64.0 ± 12.3) with diverse etiologies such as stroke, parkinsonism, or vagal palsy. Random numbers were given for the VFSS images of 10 patients and distributed to two experienced examiners to assure blindness. The swallowing motions were analyzed in this evaluation using the motions of the hyoid bone, larynx, epiglottis, arytenoid, upper esophageal sphincter, and food bolus.

**Fig. 4:**
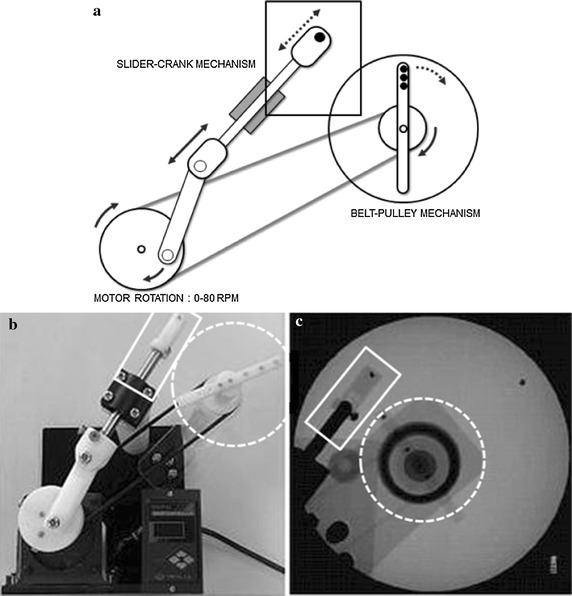
Diagram (**a**), real, and fluoroscopic images (**b**, **c**) of the instrumental swallowing model to mimic the movement of the hyoid bone and the epiglottis using the slider–crank and the belt–pulley mechanism (Adapted from [12])

For the validity tests of the measurements of linear and angular displacements, several objects and figures were used (e.g., coins and bars with various sizes and figures of different angles of which the reference values were previously determined). We analyzed 14 instruments of the different size from 6.22 to 48.64 mm and 15 figures of the different angle from 10° to 160°. An additional file shows the information about these instruments and figures in more detail (see Additional file 1). For the validity tests of the measurements of the linear and angular velocities, we used the instrumental swallowing model which had been designed and developed to mimic the movement of the hyoid bone and the epiglottis in the previous study. The readers are encouraged to consult a previous work for the detailed explanations of the instrument [12]. The model consisted of a slider–crank and a belt–pulley mechanism (Fig. 4). We analyzed 19 video files containing different linear and angular motions using this software.

### Statistical analysis

The intraclass correlation coefficient (ICC) between the two measurements was calculated to evaluate the intra- and interrater reliabilities. The ICC value is excellent when it is greater than 0.75, good when between 0.60 and 0.74, moderate when between 0.40 and 0.59, and poor when below 0.40 [13]. The Pearson correlation coefficient and the Bland–Altman plots were acquired between the measurements and the reference values to prove the software validity. Statistical significance was set at P < 0.05. Data analysis was conducted using SPSS software (v21.0, IBM, Armonk, NY, USA).

## Results and discussion

### Reliability and validity of the program

Table 1 shows the results of the inter- and intrarater reliability tests. For the displacement of the swallowing structures, the intrarater reliability test showed an excellent agreement with the ICC values ranging from 0.87 to 0.99. The interrater reliability test also resulted in an excellent agreement with the ICC values between 0.86 and 0.98. For the velocities of the swallowing structures, the intrarater reliability indicated a moderate to excellent agreement from 0.55 to 0.99, while the interrater reliability exhibited a good to excellent agreement from 0.67 to 0.98.

**Table 1:** The results of intra- and interrater reliability tests for the displacement and velocity of the swallowing structures

Parameter	Intrarater reliability			Interrater reliability		
	ICC	95% CI		ICC	95% CI	
		Lower	Upper		Lower	Upper
Hyoid bone, maximal displacement (mm)						
Vertical	0.981	0.924	0.995	0.928	0.710	0.982
Horizontal	0.990	0.962	0.998	0.972	0.886	0.993
Two dimensional	0.979	0.914	0.995	0.948	0.790	0.987
Hyoid bone, maximal velocity (mm/s)						
Vertical	0.982	0.926	0.995	0.922	0.686	0.981
Horizontal	0.990	0.960	0.998	0.984	0.937	0.996
Two dimensional	0.949	0.796	0.987	0.960	0.839	0.990
Larynx, maximal displacement (mm)						
Vertical	0.959	0.836	0.990	0.860	0.438	0.965
Horizontal	0.869	0.471	0.967	0.893	0.571	0.974
Two dimensional	0.947	0.785	0.987	0.965	0.858	0.991
Larynx, maximal velocity (mm/s)						
Vertical	0.939	0.755	0.985	0.809	0.231	0.953
Horizontal	0.701	− 0.202	0.926	0.666	− 0.346	0.917
Two dimensional	0.946	0.782	0.987	0.797	0.183	0.950
Arytenoid, maximal displacement (mm)						
Vertical	0.883	0.528	0.971	0.895	0.577	0.974
Horizontal	0.855	0.418	0.964	0.767	0.060	0.942
Two dimensional	0.927	0.707	0.982	0.905	0.617	0.976
Epiglottis, maximal tilt angle (degree)						
From the initial position	0.906	0.622	0.977	0.868	0.468	0.967
From the y axis	0.889	0.554	0.972	0.886	0.539	0.972
Epiglottis, maximal angular velocity (degree/s)	0.554	− 0.795	0.889	0.680	− 0.290	0.920
Upper esophageal sphincter opening, distance (mm)	0.988	0.951	0.997	0.980	0.919	0.995
Liquid bolus, maximal velocity (mm/s)	0.988	0.953	0.997	0.924	0.692	0.981

The Pearson correlation coefficients between the measured and instrumental reference values were nearly 1.00 (*P* < 0.001) for the linear/angular displacements and the velocities. The Bland–Altman plots showed good agreement between the measurements and the reference values, with most estimates falling within 95% limits of agreement. The mean bias and the 95% limits of agreement were − 0.07 and − 0.45 to 0.31 mm for the linear displacement, 0.29 and − 1.59° to 2.18° for the angular displacement, 0.79 and − 5.25 to 6.83 mm/s for the linear velocity, and − 1.57 and − 5.45° to 2.31°/s for the angular velocity, respectively. The Bland–Altman plots of these values were provided in the additional file (see Additional files 2, 3).

### Case study

We presented a sample SKA with an example video clip. The AVI video clip contained a single swallow of a healthy adult obtained from a VFSS. The analysis began with settings in the preference dialog. First, we chose the structures of concern. We selected the hyoid bone, epiglottis, and food bolus as the target structures in this analysis. The motion start and end were marked as “bolus cross the mandible angle” and “bolus tail cross the UES” respectively in the event table view to obtain the pharyngeal transit time. We used a coin with 24 mm diameter as the reference object to calculate the scale factor. The number of frames used to measure the coin length was set to three. We then opened the video clip, and a main workspace window opened with it.

The main window showed the first frame of the clip after the video file was opened. The frame number was shown at the top of the right side (frame explorer) (Fig. 2). The swallowing structures we selected in the preference dialog were checked in the second column of the marker table view. The arrow in the first column indicated the structure to be marked with the next mouse click. By default, the coin was the first target of marking, followed by the hyoid bone, epiglottis, a head of bolus, and cervical 2 and 4 vertebral bodies. The marking was conducted frame-by-frame for all frames. Copying the positions of the markers from the previous frame was possible using the checkbox “Copy markers to new frame” if the marker positions were not changed between the previous and current frames. The data set during the work can be saved using the “Save Data As…” button. The “Load Data…” button lets the program retrieve the data set from a mat-file.

After digitization process was completed, it was possible to export the analyzed data as a plain text file format using the “Export…” button. The analyzed data for the selected swallowing structures were recorded as numerical values, including linear/angular displacements and velocities.

Visualization is categorized into three types as follows: the array of plots for the linear/angular displacements and velocities, a superimposed graph, and an animation of the target markers (Fig. 3). The smoothing method can be determined using the “Smoothing…” button before the acquisition of the array of plots. In this analysis, the “Moving average” was selected as the smoothing method, and the span parameter was set to be 5, which is the default value for the moving average. The array of the plots and the superimposed graph can be displayed for each selected swallowing structure using the “Plot…” and “Superimposing Plot…” buttons after setting the smoothing method. The animation of target markers can also be displayed using the “Animated Movement…” button. The target markers for animation can simultaneously be chosen for single or multiple structures, including the mandible, maxilla, hyoid, epiglottis, larynx, arytenoid, upper esophageal sphincter, and head of bolus. The target order during the target marker animation is displayed for each of the ten targets. Hence, the relative speed of the moving target can be grossly observed.

### Limitations of the STAMPS software

A few precautions should be considered before the application of this tool. First, the outcome of the analyses using this software depends on the image quality of the VFSS. The swallowing structures (e.g., laryngeal cartilage) may be nearly invisible depending on the radiation dose or sampling frequency, and the degradation has a significant effect on the accuracy or consistency of the analysis. The authors are implementing image pre-processing to enhance the clarity of faint VFSS images. Second, the sampling frequency of video files is a significant factor influencing the finite difference approximation of velocities. The accuracy or consistency is poorer than expected if the sampling frequency is not high enough to analyze fast swallowing motions.

## Conclusions

This study presented the swallowing motion analysis software, STAMPS which has good reliability and validity with a user-friendly graphic interface. It is expected to be applied for research or clinical settings as a practical means to evaluate the swallowing status or treatment efficacy. STAMPS currently requires MATLAB, which is one of the most popular and reliable platforms in numerical and symbolic analyses. The software presented herein is open source and researchers can add or modify it according to their research needs. The only drawback of the software is the expensive license fee of MATLAB. Considering this limitation, the authors are considering an alternative platform (e.g., Python) to resolve the problem.

## Additional files

**Additional file 1: Figure S1.** Fluoroscopic images of different lengths and angles for validation. The lengths (A) and angles (B) were measured by swallowing kinematic analysis and compared with predetermined reference values.

**Additional file 2: Figure S2.** The Bland-Altman plot for the lengths (A) and angles (B). The graph shows the relation between the mean of the two values (reference and STAMPS value, the x-axis) and the difference between the two (the y-axis).

**Additional file 3: Figure S3.** The Bland-Altman plot for the linear (A) and angular (B) velocities. The graph shows the relation between the mean of the two values (reference and STAMPS value, the x-axis) and the difference between the two (the y-axis).

## Abbreviations

SKA: swallowing kinematic analysis
VFSS: videofluoroscopic swallowing study
STAMPS: spatio-temporal analyzer for motion and physiologic study
UES: upper esophageal sphincter
ICC: intraclass correlation coefficient
