# Supplementary figures and images for: STAMPS: development and verification of swallowing kinematic analysis software
Source: Biomed Eng Online. 2017 Oct 17;16:120. doi: 10.1186/s12938-017-0412-1 (PMC5645924; doi:10.1186/s12938-017-0412-1)

## Slide 1
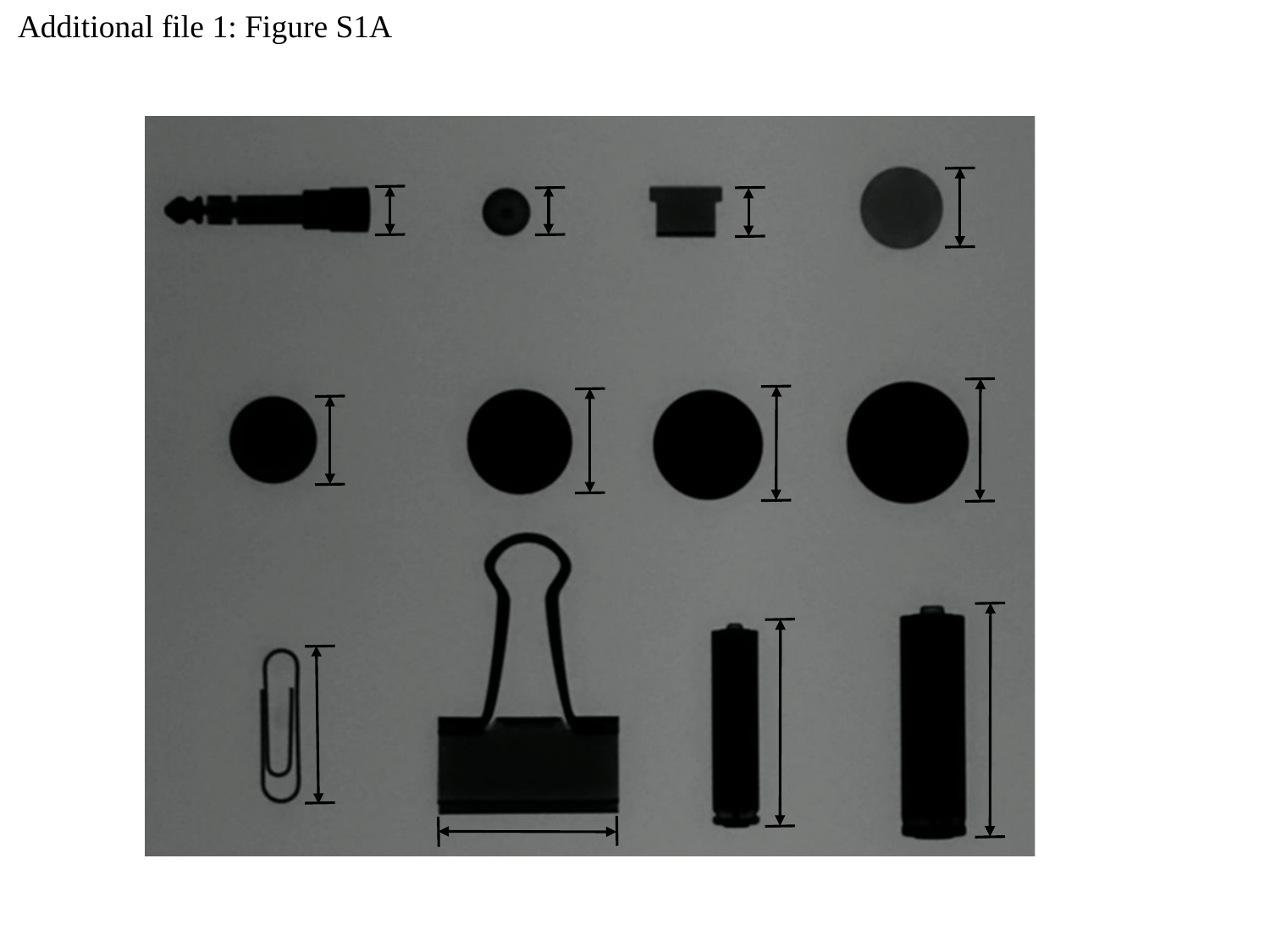

Additional file 1: Figure S1A
#

## Slide 2
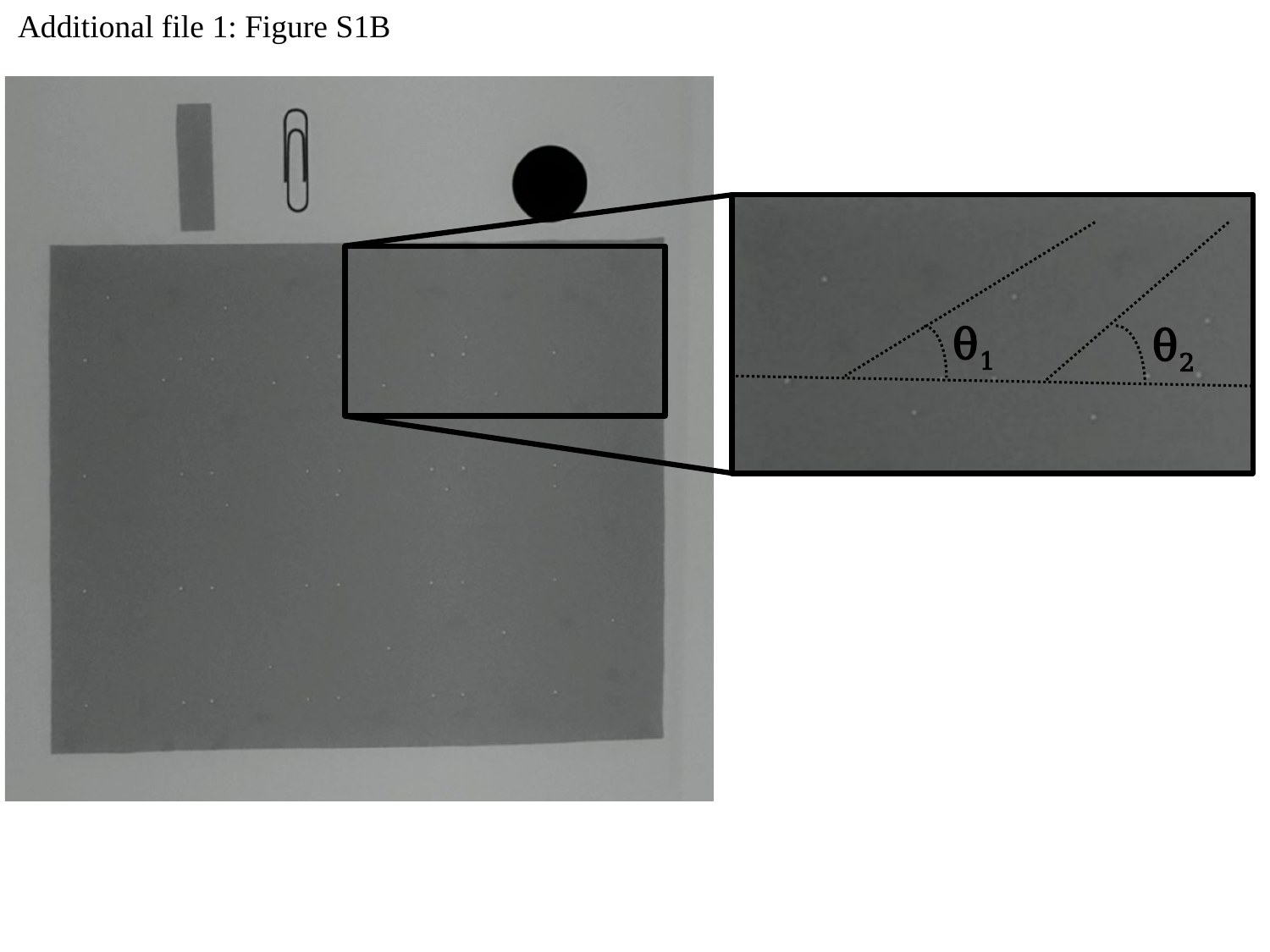

Additional file 1: Figure S1B
#
θ1
θ2

Supplement: Supplementary file 1 — Additional file 1: Figure S1. Fluoroscopic images of different lengths and angles for validation. The lengths (A) and angles (B) were measured by swallowing kinematic analysis and compared with predetermined reference values. [file 12938_2017_412_MOESM1_ESM.pptx]

## Slide 1
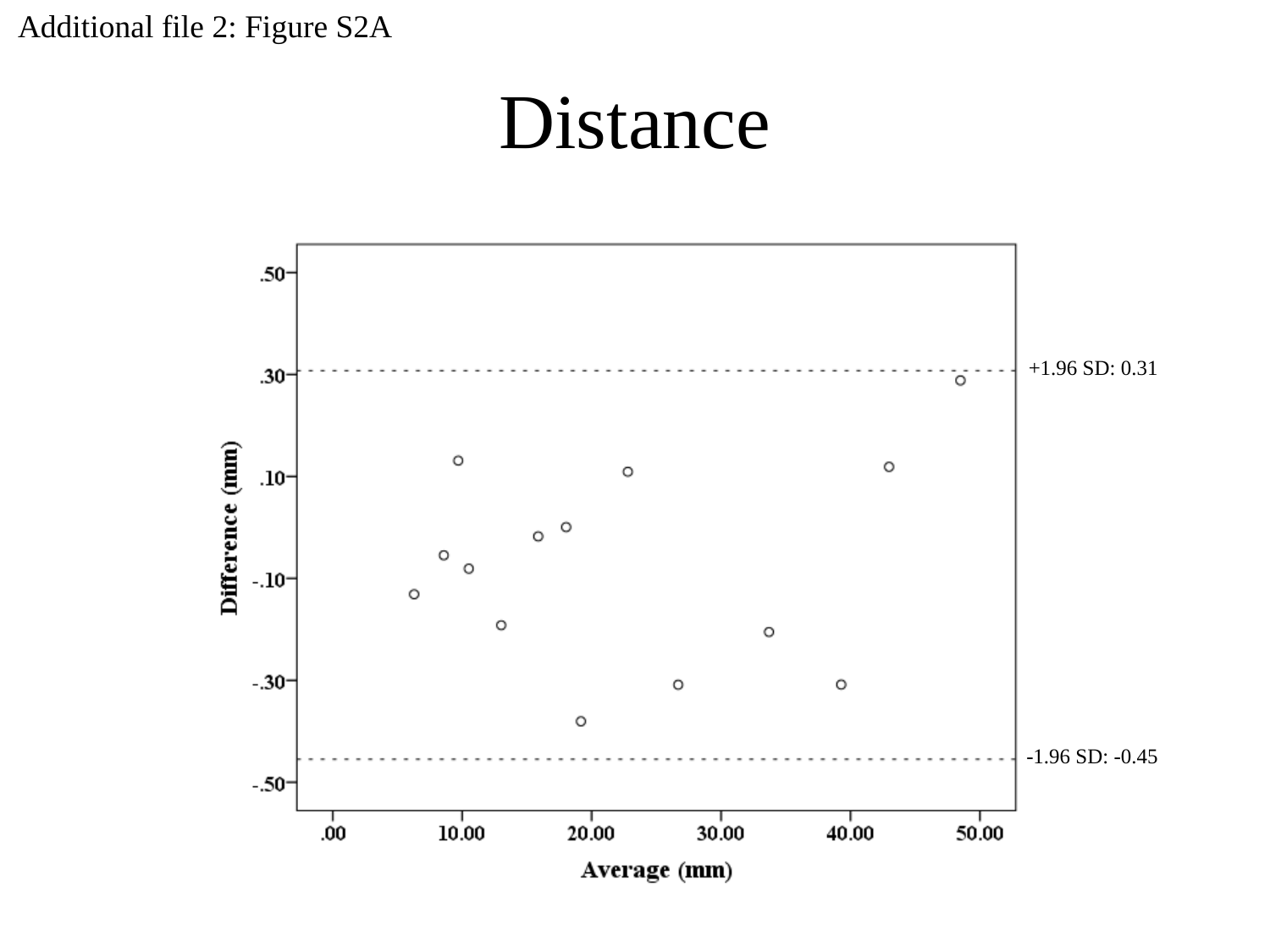

Additional file 2: Figure S2A
# Distance
+1.96 SD: 0.31
-1.96 SD: -0.45

## Slide 2
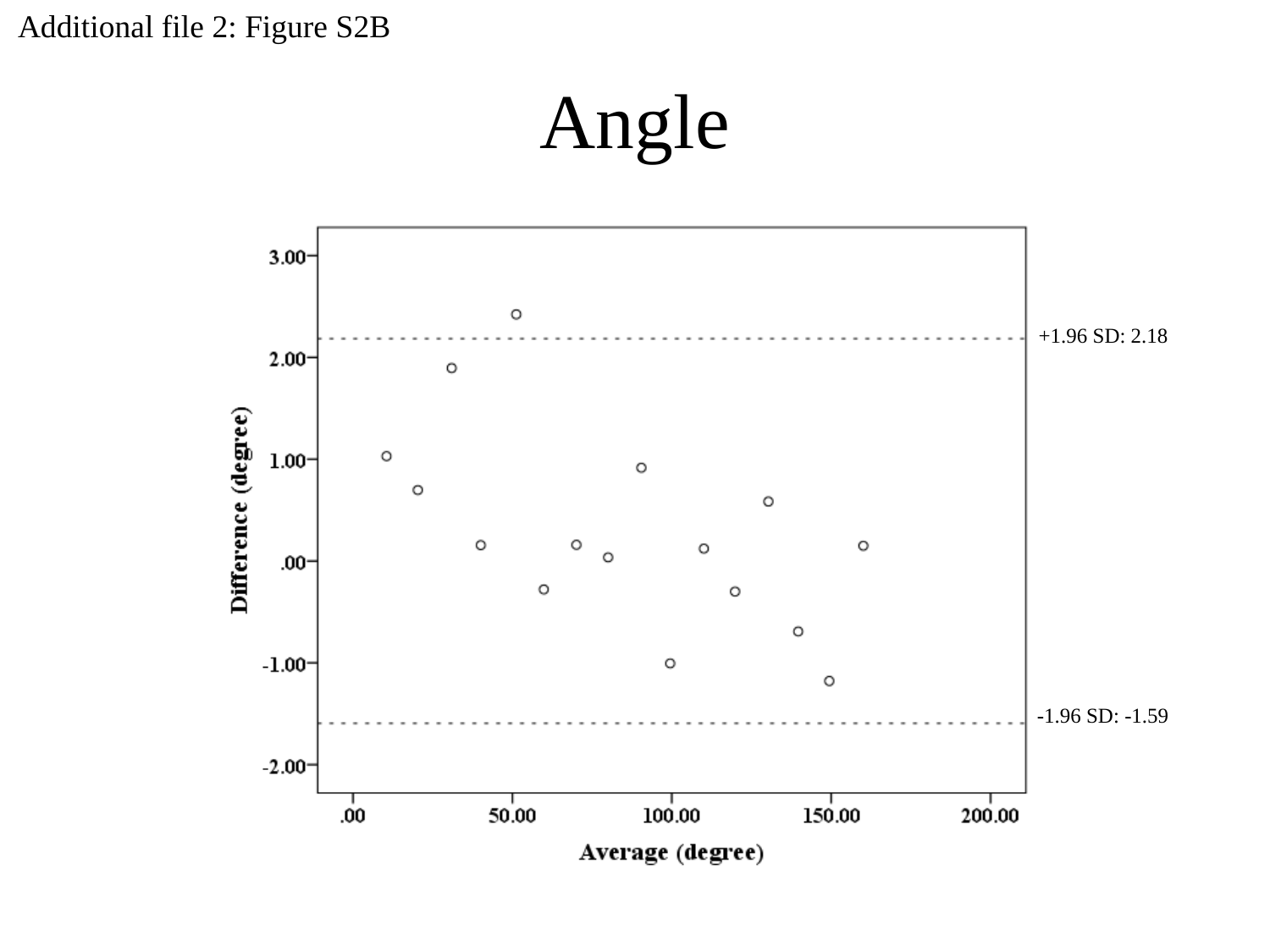

Additional file 2: Figure S2B
# Angle
+1.96 SD: 2.18
-1.96 SD: -1.59

Supplement: Supplementary file 2 — Additional file 2: Figure S2. The Bland-Altman plot for the lengths (A) and angles (B). The graph shows the relation between the mean of the two values (reference and STAMPS value, the x-axis) and the difference between the two (the y-axis). [file 12938_2017_412_MOESM2_ESM.pptx]
