# Supplementary material for: STAMPS: development and verification of swallowing kinematic analysis software
Source: Biomed Eng Online. 2017 Oct 17;16:120. doi: 10.1186/s12938-017-0412-1 (PMC5645924; doi:10.1186/s12938-017-0412-1)
Supplement: Supplementary file 3 — Additional file 3: Figure S3. The Bland-Altman plot for the linear (A) and angular (B) velocities. The graph shows the relation between the mean of the two values (reference and STAMPS value, the x-axis) and the difference between the two (the y-axis). [file 12938_2017_412_MOESM3_ESM.pptx]

## Slide 1
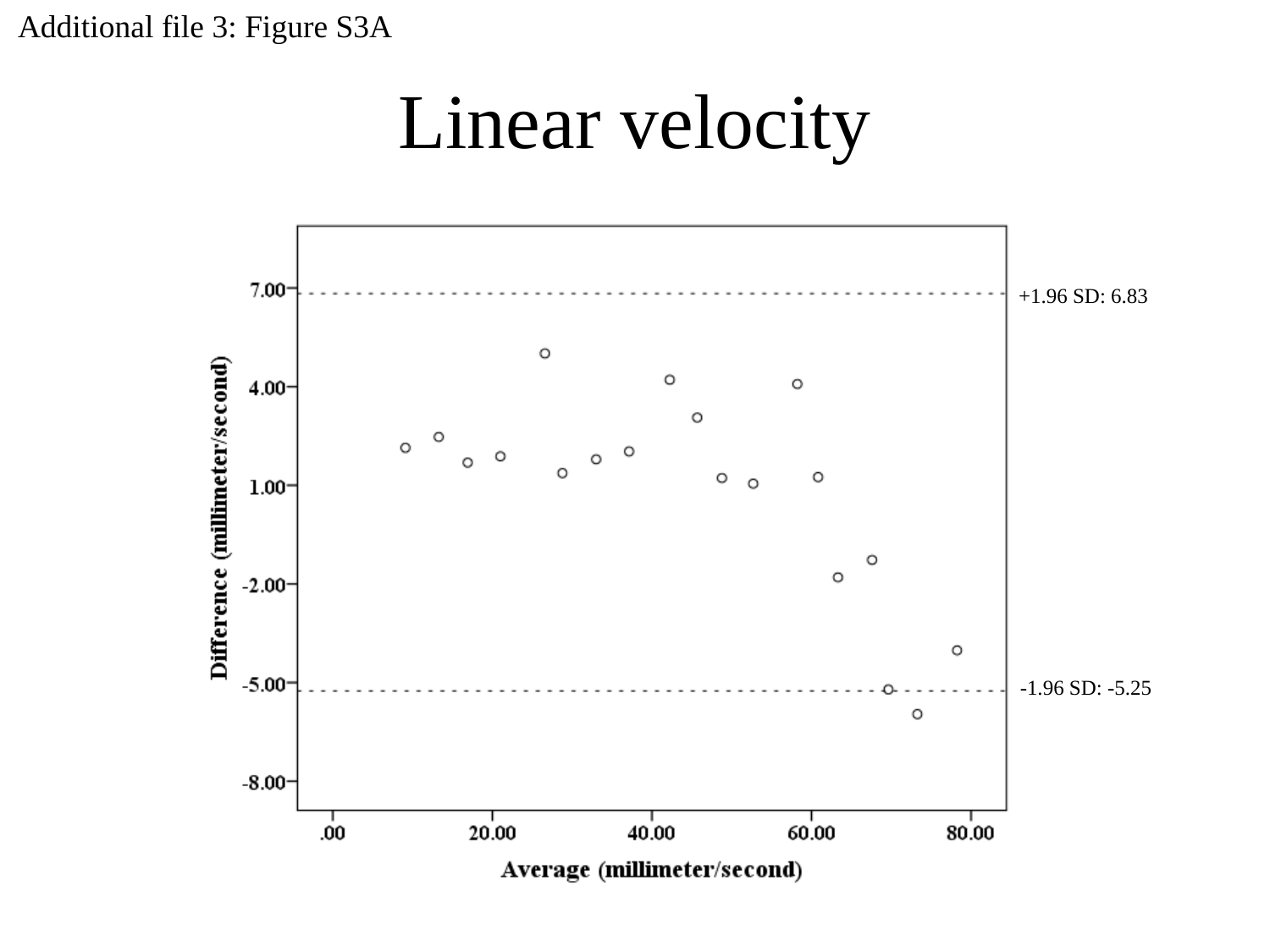

Additional file 3: Figure S3A
# Linear velocity
+1.96 SD: 6.83
-1.96 SD: -5.25

## Slide 2
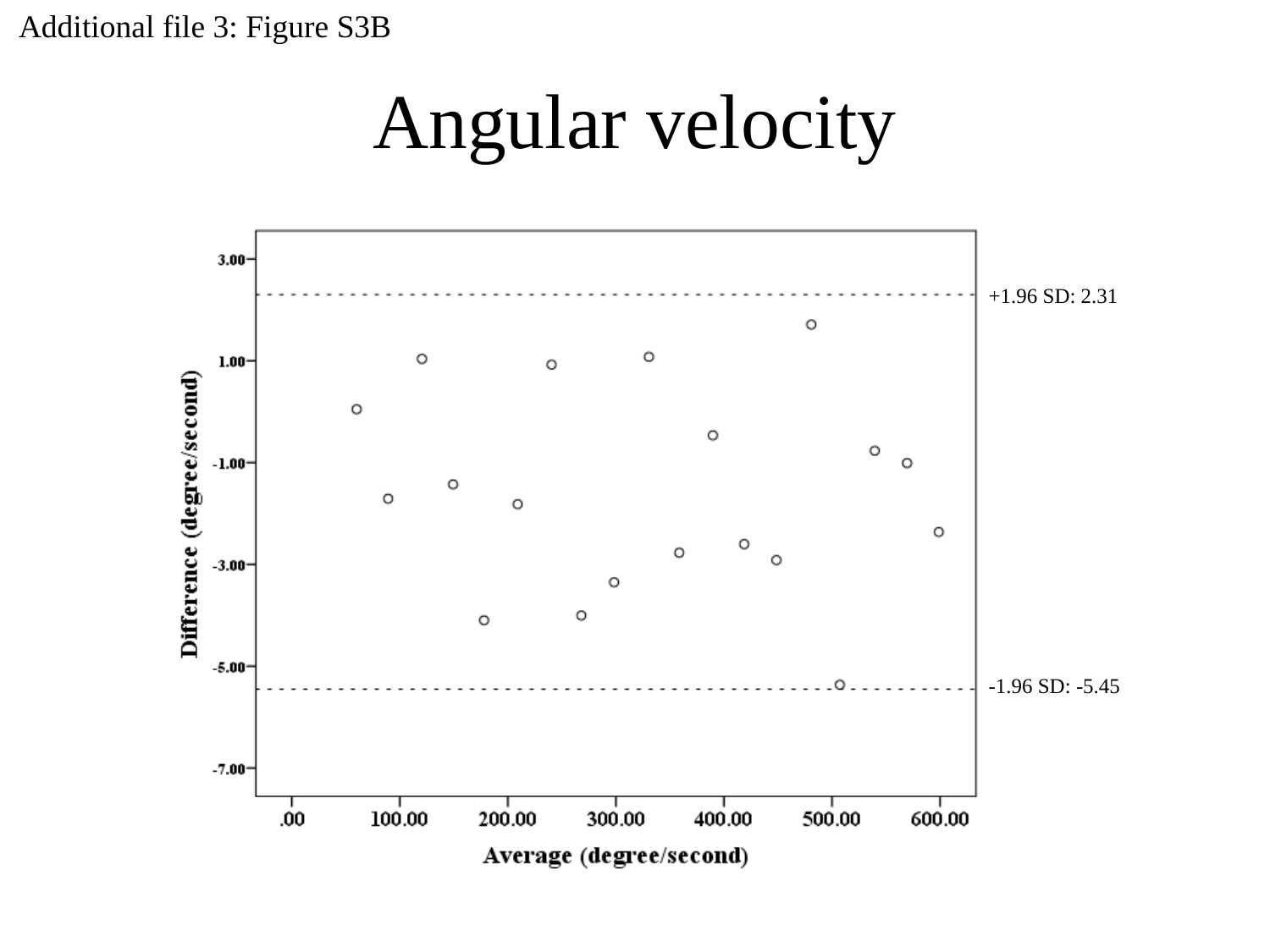

Additional file 3: Figure S3B
# Angular velocity
+1.96 SD: 2.31
-1.96 SD: -5.45
